# Deep Brain Stimulation for *VPS16* ‐Related Dystonia: A Multicenter Study

**DOI:** 10.1002/ana.27290

**Published:** 2025-06-20

**Authors:** Tatiana Svorenova, Luigi M. Romito, Ahmet Kaymak, Eoin Mulroy, Laura Cif, Elena Moro, Kirsten E. Zeuner, Simone Zittel, Jan Niklas Petry‐Schmelzer, Doreen Gruber, Liesanne Centen, Alberto Albanese, Miriama Ostrozovicova, Vladimir Han, Veronika Magocova, Kamil Knorovsky, Aurelia Kollova, Barbara Garavaglia, Nico Golfrè‐Andreasi, Chiara Reale, Alberto Mazzoni, Giovanna Zorzi, Roberto Eleopra, Vincenzo Levi, Thomas Foltynie, Patricia Limousin, Harith Akram, Ludvic Zrinzo, Francesca Magrinelli, David Murphy, Henry Houlden, Manju A. Kurian, Claudio Baiata, Steffen Paschen, Katja Lohmann, Jens Volkmann, Wolfgang Hamel, Michael T. Barbe, Martje E. van Egmond, MAJ Tijssen, Lubos Ambro, Veronika Jurkova, Robert Jech, Petra Havrankova, Juliane Winkelmann, Michael Zech, Matej Skorvanek

**Affiliations:** ^1^ Department of Neurology P.J. Safarik University Kosice Slovak Republic; ^2^ Department of Neurology University Hospital of L. Pasteur Kosice Slovak Republic; ^3^ Parkinson and Movement Disorders Unit Fondazione IRCCS Istituto Neurologico Carlo Besta Milan Italy; ^4^ The BioRobotics Institute Scuola Superiore Sant' Anna Pisa Italy; ^5^ Unit of Functional Neurosurgery, Department of Clinical and Movement Neurosciences UCL Queen Square Institute of Neurology London UK; ^6^ Department of Neurosurgery, Gui de Chauliac Hospital University Hospital Montpellier Montpellier France; ^7^ Service of Neurology, Department of Clinical Neurosciences Lausanne University Hospital (CHUV) and University of Lausanne (UNIL) Lausanne Switzerland; ^8^ Grenoble Alpes University, Division of Neurology, CHU of Grenoble Grenoble Institute of Neurosciences Grenoble France; ^9^ Department of Neurology Universitätsklinikum Schleswig‐Holstein Kiel Germany; ^10^ Department of Neurology University Medical Center Hamburg‐Eppendorf Hamburg Germany; ^11^ Department of Neurology, Faculty of Medicine and University Hospital Cologne University of Cologne Cologne Germany; ^12^ Movement Disorders Hospital Beelitz‐Heilstätten Germany; ^13^ Department of Neurology University Medical Center Groningen, University of Groningen Groningen The Netherlands; ^14^ Expertise Center Movement Disorders Groningen University Medical Center Groningen, University of Groningen Groningen the Netherlands; ^15^ Department of Neurology IRCCS Fondazione Mondino Pavia Italy; ^16^ Laboratory of Clinical Neurosciences, University Science Park MEDIPARK P. J. Safarik University Kosice Slovak Republic; ^17^ Department of Neurosurgery P.J. Safarik University Kosice Slovak Republic; ^18^ Department of Neurosurgery University Hospital of L. Pasteur Kosice Slovak Republic; ^19^ Unit of Medical Genetics and Neurogenetics Fondazione IRCCS Istituto Neurologico Carlo Besta Milan Italy; ^20^ Department of Child Neurology Fondazione IRCCS Istituto Neurologico Carlo Besta Milan Italy; ^21^ Neurosurgery Department, Functional Neurosurgery Unit, Fondazione IRCCS Istituto Neurologico Carlo Besta Milan Italy; ^22^ Department of Clinical and Movement Neurosciences, UCL Queen Square Institute of Neurology University College London London UK; ^23^ Department of Neuromuscular Diseases, UCL Queen Square Institute of Neurology University College London London UK; ^24^ Developmental Neurosciences, Zayed Centre for Research into Rare Disease in Children UCL Great Ormond Institute of Child Health London UK; ^25^ Department of Neurology Great Ormond Street Hospital for Children London UK; ^26^ Division of Neurology CHU of Grenoble Grenoble France; ^27^ Institute of Neurogenetics University of Luebeck Luebeck Germany; ^28^ Department of Neurology University of Würzburg Würzburg Germany; ^29^ Department of Neurosurgery University Medical Center Hamburg‐Eppendorf Hamburg Germany; ^30^ Center for Interdisciplinary Biosciences, Technology and Innovation Park P.J. Safarik University Kosice Slovak Republic; ^31^ Department of Health Psychology and Research Methodology, Faculty of Medicine P.J. Safarik University Kosice Slovak Republic; ^32^ Department of Neurology, Charles University in Prague 1st Faculty of Medicine and General University Hospital in Prague Praha, Prague Czech Republic; ^33^ Institute of Neurogenomics Helmholtz Zentrum München Munich Germany; ^34^ Institute of Human Genetics, School of Medicine Technical University of Munich Munich Germany; ^35^ Munich Cluster for Systems Neurology, SyNergy Munich Germany; ^36^ Institute for Advanced Study Technical University of Munich Garching Germany

## Abstract

**Objective:**

The objective was to evaluate the effects of deep brain stimulation (DBS) in an international cohort of patients with *VPS16*‐related dystonia.

**Methods:**

This observational study collected preoperative and postoperative demographic, clinical, stimulation, genetic, neuroimaging, and neurophysiological data of medically refractory DYT‐*VPS16* patients with implanted DBS in 10 international centers. Motor symptoms and disability outcomes were assessed using the Burke‐Fahn‐Marsden Dystonia Rating Scale Motor (BFMDRS‐M) and Disability (BFMDRS‐D) scales. A cut‐off threshold for considering response to DBS was set at 25% of BFMDRS‐M improvement at the last follow‐up (FU) compared to baseline.

**Results:**

The cohort consisted of 26 participants (17 men, 65.4%). Age at dystonia onset and surgery was 17.8 ± 10.9 and 35.3 ± 14.8 years, respectively. At the last FU, 102.5 ± 57.3 months (range, 2–216), the mean BFMDRS‐M improvement was 41.6 ± 37.3% (26/26 patients) and 34.8 ± 42.6% for the BFMDRS‐D (23/26 patients). Most patients (19/26, 73%) were considered responders. Higher motor improvement was associated with stimulation of the ventroposterior portion of the internal globus pallidus. A significant inverse relationship was observed between improvement in BFMDRS‐M at last FU, and the presence of spasticity (*p* = 0.027) and fixed skeletal deformities (*p* = 0.001) before surgery. Non‐responders had a younger age at disease onset and at implantation, shorter disease duration at DBS surgery, and higher baseline BFMDRS scores.

**Interpretation:**

DBS was an effective treatment for three‐quarters of patients with pathogenic *VPS16* variants in our cohort. Mean motor improvement was most pronounced at the 1‐year FU, but persisted at the last FU despite disease progression. ANN NEUROL 2025;98:711–725

Pathogenic *VPS16* gene variants have been recently identified as an important cause of early‐onset dystonia with a global disease burden.^1^, ^2^, ^3^, ^4^, ^5^ Although initially considered an autosomal recessive disorder,^3^ several families with autosomal dominant pattern and incomplete penetrance or cases with a de novo occurrence have been identified.^1^, ^2^, ^4^, ^5^, ^6^, ^7^, ^8^, ^9^, ^10^, ^11^, ^12^, ^13^ DYT‐*VPS16* dystonia typically presents with isolated generalized dystonia, with craniocaudal gradient, occasionally accompanied by myoclonus,^5^, ^8^, ^9^, ^13^ neuropsychiatric features, such as emotional lability, anxiety or depression,^1^, ^6^, ^9^ and intellectual disability.^1^, ^6^

Deep brain stimulation (DBS) is an effective treatment for medically refractory segmental or generalized dystonia. Predicting DBS success at an individual patient level for dystonia is difficult, but improved understanding of the genetic background of dystonia has allowed incorporation of genetic data into predictive algorithms.^14^, ^15^ In this regard, increasing evidence suggests both short‐ and long‐term DBS efficacy in a significant proportion of patients with monogenic dystonia because of *TOR1A* mutations,^14^, ^16^, ^17^ as well as in DYT‐*SGCE*,^18^ DYT/PARK‐*TAF1*,^19^ DYT‐*KMT2B*,^20^ DYT‐*GNAL*,^21^ or DYT‐*GNAO1* dystonia.^22^, ^23^ Moreover, previous reports show minimal or no improvement after DBS in patients with DYT‐*ATP1A3* dystonia.^23^, ^24^ Although several previous reports suggest a potential benefit of DBS also in DYT‐*VPS16* dystonia, current evidence has not been sufficient to draw final conclusions for clinical decision‐making.^23^

The aim of our study was to evaluate short‐ and long‐term effects of DBS in an international retrospective cohort of patients with *VPS16*‐related dystonia. We also aimed to evaluate factors associated with good versus poor outcomes in these patients, including genetic variants, clinical factors, and lead positions, as well as to describe pallidal neuronal activity acquired during perioperative microelectrode recordings in this clinico‐genetic syndrome.

## Methods

### Recruitment and Ethical Approval

Thirty‐seven centers from Europe, Americas, Asia, and Australia were contacted for the availability of patients with DYT‐*VPS16* dystonia and implanted DBS (see Supporting Information). In total, 26 patients with available data were identified from 10 DBS centers in 6 European countries, including Grenoble, Montpellier (both France), Berlin, Cologne, Hamburg, Kiel (all Germany), Milan (Italy), Groningen (Netherlands), Kosice (Slovakia), and London (United Kingdom). Inclusion criteria were as follows: (1) genetically confirmed DYT‐*VPS16* dystonia, treated with DBS, (2) at least 1 available follow‐up (FU) more than 1 month after the surgery and Burke‐Fahn‐Marsden Dystonia Rating Scale (BFMDRS) motor sub‐score available at least at baseline before surgery and at last FU.

Central ethical approval for this study was obtained by the ethics committee of the University Hospital of L. Pasteur in Košice, Slovak Republic, under no. 2022/EK/11088. The study was performed according to the Declaration of Helsinki. Informed consent of all participating patients was provided by each center individually. The methodology followed the STROBE guidelines for a cohort study.

### Genetic Data

Genetic data were collected for each patient. For each variant, we determined whether variants were previously described, reported on mutation databases, or novel and were classified according to the American College of Medical Genetics and Genomics (ACMG) guidelines.^25^ Loss‐of‐function variants were classified as pathogenic. Missense variants were further evaluated based on Combined Annotation Dependent Depletion (CADD) score and other in‐silico prediction programs (PolyPhen‐2, SIFT, Provean, and MutationTaster) as well as frequency in the Genome Aggregation Database (gnomAD). Missense changes that were predicted to be deleterious by at least 3 tools and that were absent from gnomAD were considered disease‐causing.

We grouped patients into 2 categories: protein‐truncating variants (start‐loss, nonsense, frameshifts, and splice‐site) or missense variants. The model of the human VPS16 protein structures was retrieved from the AlphaFold Protein Structure Database (AlphaFold DB) by their Uniprot accession code.^26^, ^27^, ^28^

### Data Collection

We collected basic demographic information as well as age of disease onset. Detailed phenotypic data were collected, including site of dystonia onset and distribution of dystonia at baseline and at the last available FU assessment. If available, also data at first FU (1–3 months), 6, and 12 months after implant were assessed. The presence or absence of spasticity, fixed skeletal deformities, neuropsychiatric symptoms, epileptic seizures, either diagnosed by relevant scales or based on treating neurologist reporting (present/not present), and information on comorbidities were also collected. Data on previous treatment and its effect on dystonia were recorded. DBS‐related information included age at surgery, DBS target, type of device, surgical complications, stimulation parameters, stimulation‐related side‐effects, and device/hardware‐related complications. Primary outcomes of the study included evaluation of dystonia as assessed by BFMDRS‐motor (M)^29^ and BFMDRS‐disability (D) subscales.^29^ The cut‐off for non‐responders was set as an improvement of BFMDRS‐M <25% at last FU. Responders with improvement more than >50% were further subclassified as high responders.^30^, ^31^ Secondary outcomes included Clinician and Patient Global Impression–Improvement Scales (CGI and PGI, respectively). Primary and secondary outcomes were recorded for each of the study timepoints as available, including baseline status before DBS surgery.

### Statistical Analysis

Statistical analysis was performed using SPSS Version 25 statistic software package. Data were expressed as mean ± standard deviation (SD) and statistical significance was set at *p*‐value <0.05. Non‐parametric tests were used if the data were not distributed normally for comparisons between groups. Improvement of BFMDRS was calculated as ([BFMDRS baseline‐FU]/baseline) × 100. Mann–Whitney *U* test was used to identify the relationship between age of disease onset and genotype and dystonia severity represented by BFMDRS‐M at baseline and type of mutation. The evolution of BFMDRS‐M at baseline and last FU with DBS according to the class of variant was analyzed by Wilcoxon signed‐rank test. The Wilcoxon signed‐rank test was used for the comparison of BFMDRS scores and PGI and CGI at each timepoint. Correlations between improvement of BFMDRS and independent continuous variables such as the age of dystonia onset, age at DBS implantation, and disease duration were identified with non‐parametric Spearman's rho test. For ordinal values (gender, fixed skeletal deformities, spasticity, intellectual disability, anxiety, depression, emotional lability, epileptic seizures, and other comorbidities), we used non‐parametric Mann–Whitney *U* test. Additionally, analysis of variance regression analysis was used to study relationships between selected significant factors and response to DBS. Differences between responders and non‐responders were characterized by eta (continuous) and phi (ordinal variables) coefficient and individually, significant factors of DBS outcome were analyzed by binomial logistic regression. The Spearman's rho test was used also for expression of relationship between subjective PGI and objective BFMDRS improvement.

### DBS Sweet and Sour Spot Mapping and Microelectrode Recordings Data Analysis

Pre‐ and post‐surgical neuroimaging data (computed tomography or magnetic resonance imaging) were collected for electrode position reconstructions. If available, data on neurophysiological perioperative microelectrode recording were also collected. We localized DBS electrodes for the patients in our cohort using the advanced processing pipeline available in Lead‐DBS v2.5.^32^ For electric fields (E‐fields) estimation, we followed the methodology published previously.^33^ To examine the impact of stimulation intensity and position on globus pallidus internus (GPi)‐DBS responsiveness in DYT‐*VPS16* patients, we applied a voxel‐wise correlation analysis.^33^, ^34^ Specifically, E‐fields were modelled in 22 patients (3 patients were excluded from this analysis because of low image quality, Table S1) based on active contacts and stimulation parameters, and were analyzed separately for the right and left hemispheres. To identify and visualize sweet and sour spot regions based on voxel‐wise correlation scores, we processed voxel data using a combination of statistical filtering and 3‐dimensional visualization techniques for each hemisphere separately as outlined in Supporting Information.^33^, ^34^

We also examined intraoperative microelectrode recordings (MER) from 5 trajectories acquired during pallidal DBS surgery under propofol anesthesia in 2 patients (P8 and P9) to characterize the neural activity of the internal and external segments of the globus pallidus (GPi and GPe, respectively). We applied Dunn's test with Holm‐Bonferroni multiple comparison correction for continuous neural features and Fisher exact test for binary neural features for GPi‐GPe comparison as described previously^35^ (see Supporting Information for more details).

## Results

### Genetic Data

**FIGURE 1:**
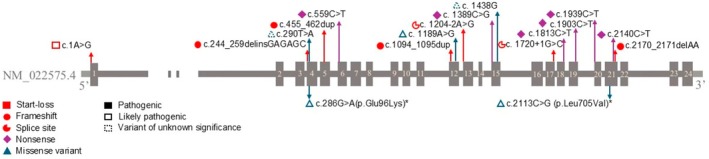
Schematic representation of *VPS16* gene (NM_022575.4) indicating the variant positions and pathogenicity according to American College of Medical Genetics and Genomics guidelines. *Compound heterozygote. [Color figure can be viewed at www.annalsofneurology.org]

We identified 26 patients with 18 different *VPS16* gene variants in our cohort, 4 of them novel (c.1A>G, c.290 T>A, c.1189A>G, and c.1204‐2A>G), not reported in mutation databases (ClinVar), and another 4 newly associated with dystonia phenotype (c.286G>A, c.2113C>G, c.1438G, and c.1813C>T). Two patients had variants of uncertain significance according to ACMG guidelines, the rest were classified as pathogenic (18/26) or likely pathogenic (5/26 and 1/26 compound heterozygote c.286G>A + c.2113C>G, P21). Most patients 22 of 26 had protein truncating variants (PTV) (1 start‐loss, 6 nonsense, 4 frameshift, and 2 splice site) 5 missense variants were identified (3/26 patients and 1/26 previously mentioned compound heterozygote, patient P21). Two families were present in the cohort, P19/P20 and P18/P16, P17, both being mother/children. Genetic background of the cohort and position of variants on gene are shown in Table 1 and Figure 1.

**TABLE 1:** Genetic Background of the Cohort (n = 26)

cDNA	p number	Patient no.	Variant type	ACMG classification
c.1A>G	p.?	2	Start‐loss	Likely pathogenic
c.1A>G	p.?	3	Start‐loss	Likely pathogenic
c.244_259delinsGAGAGC	p.K82Efs*124	23^7^	Frameshift	Pathogenic
c.286G>A + c.2113C>G	p.Glu96Lys; p.Leu705Val	21	Missense; missense	Likely pathogenic; likely pathogenic
c.290 T>A	p.Leu97Gln	26	Missense	Variant of uncertain significance
c.455_462dup	p.Leu155Alafs*59?	25^1^	Frameshift	Pathogenic
c.559C>T	p.Arg187*	1	Nonsense	Pathogenic
c.559C>T	p.Arg187*	7^6^	Nonsense	Pathogenic
c.559C>T	p.Arg187*	**8** ^6^	**Nonsense**	**Pathogenic**
c.559C>T	p.Arg187*	9^1^, ^6^	Nonsense	Pathogenic
c.1094_1095dup	p.Tyr366Serfs*12	19^1^	Frameshift	Pathogenic
c.1094_1095dup	p.Tyr366Serfs*12	20^1^	Frameshift	Pathogenic
c.1189A>G	**p.Lys397Glu**	**5**	**Missense**	**Likely pathogenic**
c.1204‐2A>G	p.?	14	Splice‐site	Likely pathogenic
c.1389C>G	p.Tyr463*	**24**	**Nonsense**	**Pathogenic**
c.1438G	**p.Gly480Ser**	**22**	**Missense**	**Variant of uncertain significance**
c.1720 + 1G>C	**p.?**	**11**	**Splice‐site**	**Likely pathogenic**
c.1813C>T	p.Arg605*	**12**	**Nonsense**	**Pathogenic**
c.1903C>T	p.Arg635*	4	Nonsense	Pathogenic
c.1903C>T	p.Arg635*	6^37^	Nonsense	Pathogenic
c.1903C>T	p.Arg635*	**10**	**Nonsense**	**Pathogenic**
c.1939C>T	p.Arg647*	16^13^	Nonsense	Pathogenic
c.1939C>T	p.Arg647*	17^13^	Nonsense	Pathogenic
c.1939C>T	p.Arg647*	18^13^	Nonsense	Pathogenic
c.2140C>T	p.Gln714*	15^13^	Nonsense	Pathogenic
c.2170_2171delAA	p.Lys724Glufs*44	13^13^	Frameshift	Pathogenic

Bold represents non‐responders (n = 7). Steel et al^1^; Ostrozovicova et al^6^; Pott et al^7^; Monfrini et al^13^; Petry‐Schmelzer et al.^37^

ACMG = American College of Medical Genetics and Genomics.

### Demographic and Clinical Characteristics at Baseline

**FIGURE 2:**
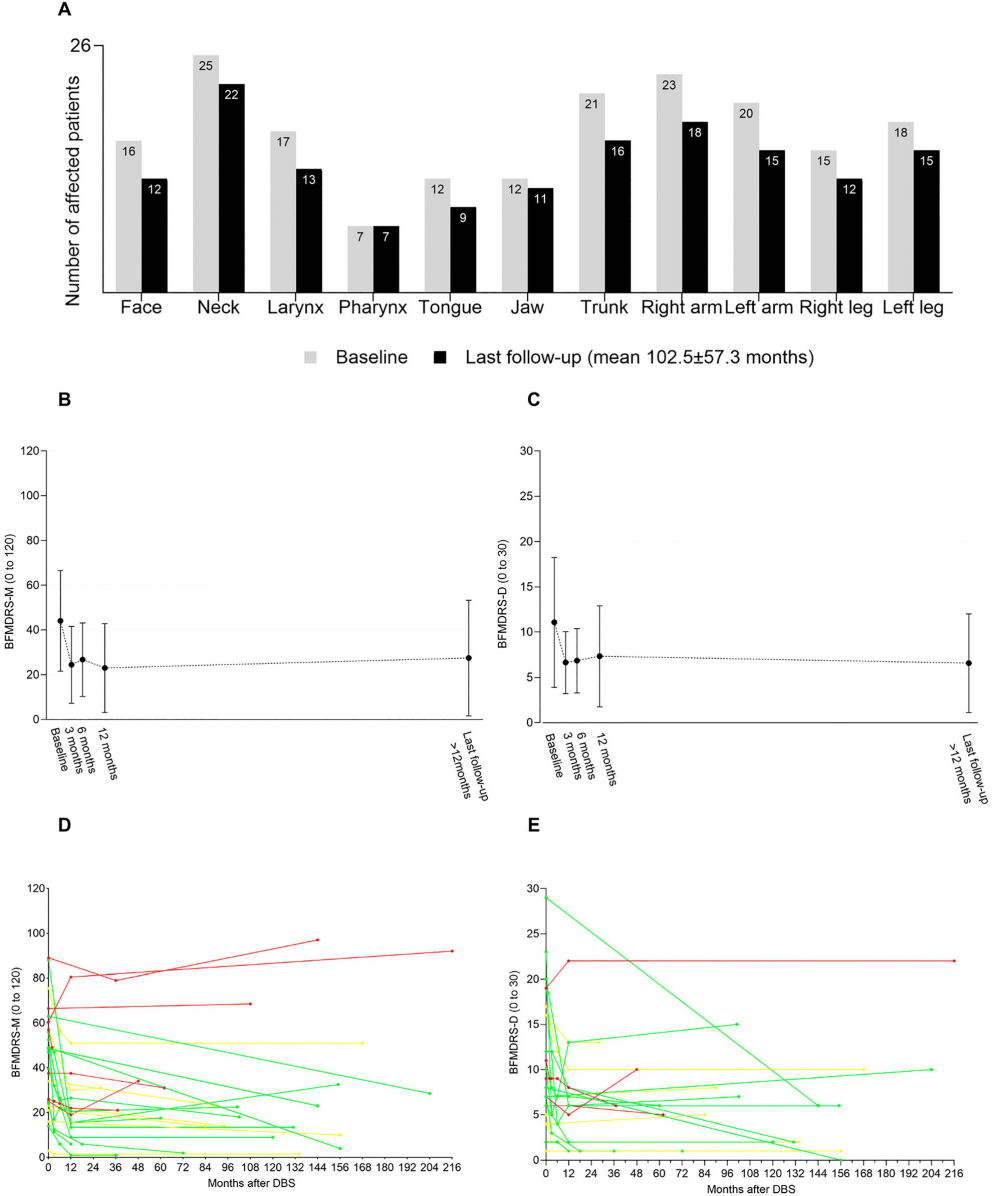
Burke‐Fahn‐Marsden Dystonia Rating Scale (BFMDRS) sub‐scores evolution and dystonia distribution before and after deep brain stimulation (DBS). (A) Distribution of dystonia of the cohort (n = 26) before DBS at baseline and at the last follow‐up, including last follow‐ups <12 months after DBS (mean 102.5 ± 57.3 months). Numbers in columns represent the number of affected patients. (B) Mean BFMDRS‐M at baseline (n = 26), 3 months (n = 8), 6 months (n = 6), 12 months (n = 16) and at the last follow‐up >12 months after surgery (mean 110.4 ± 52.0 months, n = 24) (C) Mean BFMDRS‐D at baseline (n = 23), 3 months (n = 8), 6 months (n = 6), 12 months (n = 15) and at the last follow‐up >12 months after surgery (mean 109.7 ± 55.1 months, n = 21) (D) Individual BFMDRS‐M sub‐scores evolution after DBS implantation (n = 26) with following color‐coding: green for high responders (>50%), yellow for responders (25–50%), red for non‐responders (<25% improvement in BFMDRS‐M at the last follow‐up compared to baseline). (E) Individual BFMDRS‐D sub‐scores evolution after DBS implantation (n = 23) with color‐coding based on improvement in BFMDRS‐M. [Color figure can be viewed at www.annalsofneurology.org]

Our cohort consists of 9 females (34.6%) and 17 males (65.4%) with *VPS16‐*related dystonia and implanted DBS. Mean age at dystonia onset was 17.8 ± 10.9 years (range, 6–50 years). Dystonia's site of onset was mostly in the limbs (12/26) or the neck (8/26). Before surgery, most of the patients had generalized dystonia except 3 cases with segmental distribution of the disease. Most of them presented with cervical dystonia (25/26), at least 1 limb (25/26, all of them including arm involvement) or trunk (21/26) involvement (Fig 2A). Four patients (Patients 13, 16, 21, 23) had combined dystonia with myoclonus and Patient 16 presented also with freezing of gait before surgery (Table 2).

**TABLE 2:** Basic Demographics and Clinical Characteristics of the Cohort

Patient no.	Gender	Age of onset (yr)	Disease duration before DBS (yr)	Last FU (months)	Site of onset	Body distribution^a^	Fixed skeletal deformities and spasticity^a^	Neuropsychiatric features^a^	Other movement disorders^b^	BFMDRS motor improvement^c^ (%)	BFMDRS disability improvement^c^ (%)
1	M	16	17	156	Neck	G		A, D		91.84	100.00
2	M	21	27	84	Neck, larynx	G		A		33.33	−25.00
3	M	8	16	204	NA	G		A, D, Em		54.76	−42.86
4	F	33	24	156	Neck	S				37.50	0.00
**5**	**F**	**8**	**34**	**144**	**ULs, LLs**	**G**	**Fx, Sp**	**Em**		**−8.99**	**NA**
6	M	33	11	36	Right UL	S		D		92.86	50.00
7	M	15	5	72	Neck, trunk	G		A		91.67	85.71
**8**	**M**	**30**	**29**	**2**	**LLs**	**G**	**Fx**	**ID, A, D, Em**		**14.04**	**18.18**
9	M	16	12	12	Neck, larynx then right UL	G		A, Em		75.51	80.00
**10**	**M**	**6**	**9**	**216**	**UL** ^**d**^	**G**	**Fx, Sp**	**ID, A, OCD**		**−52.07**	**−15.79**
**11**	**M**	**8**	**11**	**48**	**LLs**	**G**				**−36.00**	**−42.86**
**12**	**F**	**15**	**13**	**37**	**Larynx**	**G**		**A, Em**		**19.23**	**33.33**
13	F	12	11	131	Jaw, larynx, tongue	G			Myo	72.45	83.33
14	M	17	24	90	Left UL tremor	G	Fx	A, Em, psychosis		32.35	−14.29
15	M	12	30	60	Larynx, tongue, jaw	G				62.77	40.00
16	M	14	35	102	Larynx, UL^d^ tremor	G		D	Myo, f	65.71	63.16
17	F	13	28	155	Left UL	G		A, D		63.07	70.00
18	F	40	28	101	Neck, trunk	G				59.82	34.78
19	F	19	15	168	Right LL	G	Fx	ID, A, Em		32.45	41.18
20	M	11	8	120	Face then neck	G		ID, D, A, Em		77.22	66.67
21	M	19	17	134	Head myoclonus	S		A, D	Myo	50.00	60.00
**22**	**F**	**8**	**3**	**62**	**Left LL**	**G**	**Fx**			**17.33**	**16.67**
23	M	16	14	144	Right UL	G			Myo	52.58	79.31
**24**	**M**	**9**	**9**	**119**	**Left UL**	**G**	**Fx**	**A, D, Em**		**−3.01**	**NA**
25	M	50	2	94	Neck	G		A, PTSD		39.13	NA
26	F	13	23	28	Neck, jaw, trunk	G	Fx			45.61	18.75

Bold represents non‐responders (n = 7).

All patients had bilateral GPi placement of electrode except Patient 14, who had unilateral GPi on the right side and Patient 21, who had bilateral GPi together with ventral intermediate nucleus electrodes.

^a^ At the baseline before DBS implantation.

^b^ Other comorbidities are specified in Tables [Link], [Link].

^c^ (BFMDRS baseline‐BFMDRS last follow‐up)/BFMDRS baseline × 100.

^d^ Side not specified.

A = anxiety; BFMDRS = Burke‐Fahn‐Marsden Dystonia Rating Scale; D = depression; DBS = deep brain stimulation; Em = emotional lability; f = freezing; F = female; FU = follow‐up; Fx = fixed skeletal deformities; G = generalized; GPi = globus pallidus internus; ID = intellectual disability; LL/s = lower limb/s; M = male; Myo = myoklonus; NA = not available; No. = number; OCD = obsessive‐compulsive disorder; PTSD = post‐traumatic stress disorder; S = segmental; Sp = spasticity; UL/s = upper limb/s; yr = year.

Mean BFMDRS motor and disability sub‐scores at baseline were 44.1 ± 22.5 and 11.3 ± 7.1 (24/26), respectively. Spasticity was reported in 2of 26 (7.7%) patients, fixed skeletal deformities in 8 of 26 (30.8%) patients. Neuropsychiatric features present at baseline included anxiety (15/26, 57.7%), depression (9/26, 34.6%), emotional lability (9/26, 34.6%), and intellectual disability (4/26, 15.4%) based on clinical assessment by the treating neurologist (present/not present), but results from relevant scales were missing in most centers. Single patients reported obsessive‐compulsive disorder, psychosis, and post‐traumatic stress disorder diagnosed by a psychiatrist (Table 2). Epileptic seizures were documented in 2 of 26 patients (7.7%).

The efficacy of oral medications was limited. Botulinum toxin was reported as effective in less than half of the patients (10/23, 43.5%) and partially or temporarily effective in one‐third (7/23, 30.4%) of injected patients. For detailed clinical characteristics of the cohort, see Table S1.

### DBS Insertion and Outcomes

All patients had electrodes implanted into GPi bilaterally except for P14, who had unilateral right GPi placement and P21, who had bilateral GPi placement together with thalamic ventral intermediate nucleus (VIM) electrodes in a single surgery with no reported complications. Mean age at DBS surgery was 35.3 ± 14.8 years (range, 11–68 years) and mean disease duration was 17.5 ± 9.7 (range, 2–35 years). The types of the DBS device implanted are listed in the Table S1.

Surgical complications were reported in 2 patients (2/26, 7.7%). P11 had slight left facial nerve deficit and P25 had a small pocket hematoma around implantable pulse generator without signs of infection. Device‐related complications and stimulation‐related adverse effects are listed in Table S1. In addition, P8 underwent explantation of the whole DBS system 11 months after surgery because of subjective ineffectiveness (non‐responder with low compliance and subsequently not recharging, at last FU 2 months after DBS BFMDRS‐M improvement was 14.0%).

Mean age at last FU was 43.6 ± 15.1 years. The FU period in our cohort varied between 2 and 216 months with a mean duration of 102.5 ± 57.3 months. There were significant improvements in BFMDRS‐M scores between baseline and first FU (n = 8, *p* = 0.012), 6 months (n = 6, *p* = 0.028), 12 months (n = 16, *p* = 0.003), and last FU group (n = 26, *p* = 0.001). Significant changes were measured in BFMDRS‐D between the baseline and first FU (n = 8, *p* = 0.027), 12 months (n = 15, *p* = 0.003), and last FU (n = 23, *p* = 0.002) assessments. Mean improvement of BFMDRS‐M and BFMDRS‐D at the time of last FU compared to baseline for the whole group was 41.6 ± 37.3% (range, −52.1 to 92.9%) and 34.8 ± 42.6% (23/26; range, −42.9 to 100%), respectively. When excluding patients with a FU period of ≤1 year (P8, P9) to evaluate long‐term effect of DBS, the mean improvement was 41.3 ± 37.7% (n = 24; range, −52.1 to 92.9%) and 33.4 ± 43.3 (n = 21; range, −42.9 to 100%) (Fig 2B, C), respectively. Individual BFMDRS sub‐scores evolution after DBS implantation is shown in Figure 2D, E. The mean CGI and PGI scores at last FU were 2.5 (n = 24) and 2.6 (n = 20), respectively. PGI was in correlation with objective last FU improvement in BFMDRS‐M *r*_*s*_ (−0.791, *p <* 0.001) and BFMDRS‐D *r*_*s*_ (−0.731, *p <* 0.001) scores compared to baseline.

New dystonic symptoms occurred in 9 of 26 patients (34.6%) at the time of last FU as compared to baseline. Seven of them were classified as spontaneous disease progression and 2 as stimulation induced dystonic side‐effects according to their treating neurologist. Nevertheless, at last FU, the overall number of body sites affected by dystonia decreased, except for number of patients with pharyngeal dystonia (7/26; 26.9%) (see Fig 2A). Number of patients with fixed skeletal deformities and spasticity remained the same at the last FU as before surgery (8/26; 30.8% and 2/26; 7.7%, respectively).

Intellectual disability was newly reported in 2 patients (6/26; 23.1%) at the time of last FU (P5, P7) compared to baseline. None of the patients developed anxiety or emotional lability after surgery at the time of the last FU. The number of patients with anxiety and emotional lability decreased from 15 of 26 (57.7%) at baseline to 11 of 26 (42.3%) (*p* = 0.046) at the last FU and from 9 of 26 (34.6%) to 5 of 26 (19.2%) (*p* = 0.046), respectively. Based on treating neurologists, reported presence of depression increased from 9 of 26 (34.6%) patients at baseline to 11 of 26 (42.3%) (*p* = 0.414) at the time of last FU.

A statistically significant negative relationship has been observed between improvement of BFMDRS‐M at last FU and fixed skeletal contractures (*p* = 0.001) and spasticity (*p* = 0.027) present before surgery. The regression analysis showed statistically significant dependency on these factors (*p* = 0.026, 95% confidence interval [CI] = (−59,736 to −4,116) and *p* = 0.047, 95% CI = (−98,318 to −0,682), respectively). The same relationship was not observed for improvement of BFMDRS‐D.

Seven cases (26.9%) were classified as non‐responders at last FU, only 1 showing no improvement of BFMDRS‐M at all post‐DBS. Improvement was transient in 3 of them and did not reach the 25% improvement threshold at last FU in 3 others (Table S1). Nineteen patients (19/26; 73.1%) responded to DBS, 12 of them (12/26; 46.2%) were defined as high responders (Table 2).

**FIGURE 3:**
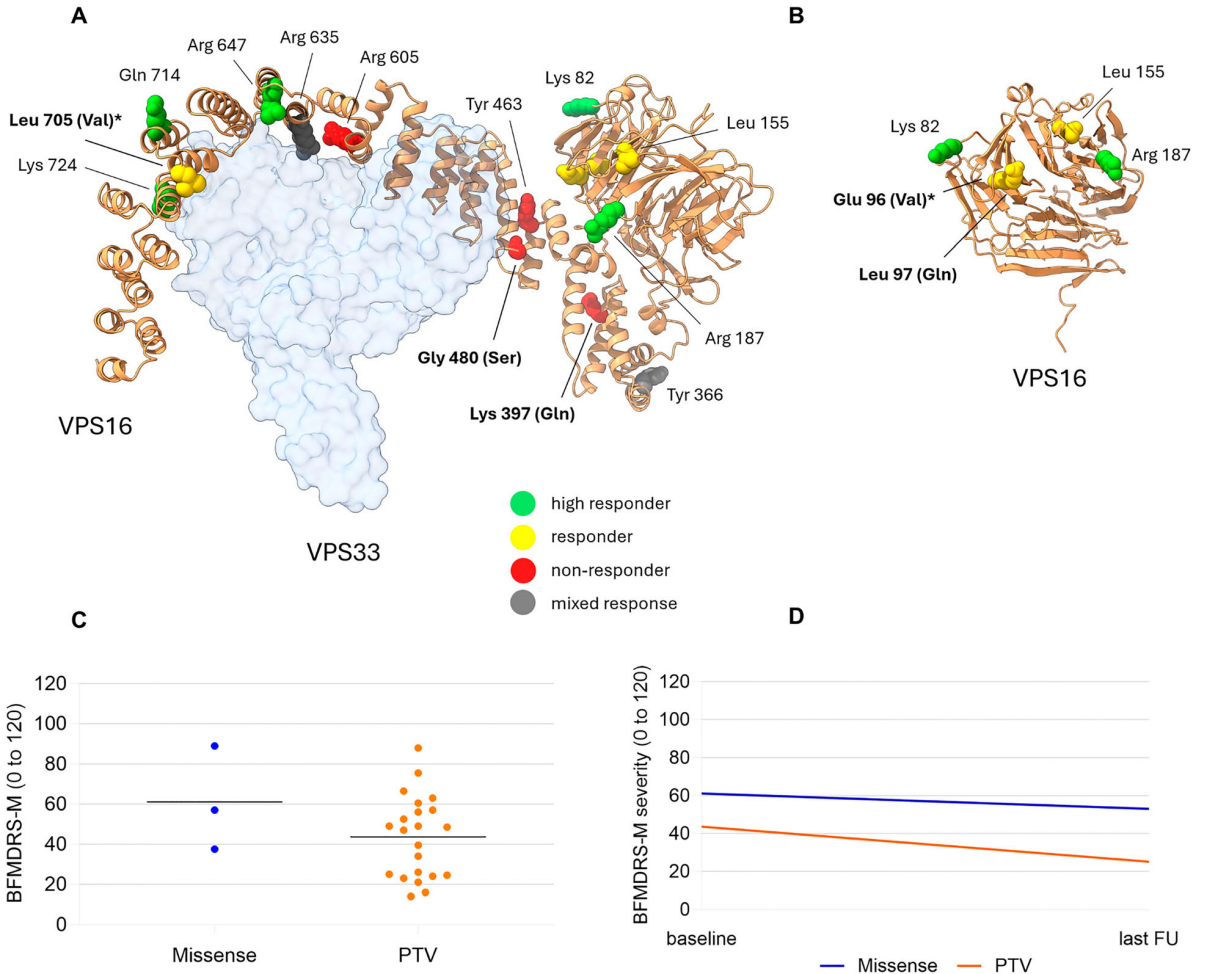
Relationship between genotype, protein position, and response to deep brain stimulation (DBS) in terms of dystonia severity. (A) Visualization of the mutations of patients with last follow‐up >12 months after DBS analyzed in this study mapped onto the AlphaFold model of the human VPS16 protein (orange ribbon, AlphaFold identifier: AF‐Q9H269‐F1). The model is overlaid with the crystal structure of a short fragment of human VPS16 (residues 642–736) in complex with human VPS33 (PDB ID: 4BX9). Target amino acid residues on VPS16 are represented as spheres, with following color‐coding: green for high responders (>50%), yellow for responders (25–50%), red for non‐responders (<25% improvement in Burke‐Fahn‐Marsden Dystonia Rating Scale‐motor (BFMDRS‐M) at the last follow‐up compared to baseline) and gray for mixed responses to DBS. The VPS33 protein is displayed as a pale blue surface, highlighting its major interaction interface with VPS16 in the human HOPS complex. Missense mutations that were further analyzed through bioinformatics predictions are labelled in bold, with the mutated amino acid in parentheses. Compound heterozygote (*), P21, was responder to DBS with 50% improvement in BFMDRS‐M. Start‐loss mutation (c.1A>G mixed responses) and splice‐site mutations (c.1204‐2A>G responder; c.1204‐2A>G non‐responder) analyzed in our cohort are not related to the position on the protein. (B) Detailed view of the β‐sheet‐rich globular domain of human VPS16, showing mutations at positions Lys 82, Glu 96, Leu 97, Leu 155, and Arg 187. This close‐up provides clarity on the spatial positions of these mutations. (C) Relationship between type of mutation and BFMDRS‐M score at baseline before DBS. Missense mutations (n = 3, blue) and protein truncating variants (PTV) (n = 22, orange). Compound heterozygote (p.Glu96Lys; p.Leu705Val), P21, was excluded from the graph (baseline BFMDRS‐M 3 points). (D) Dystonia BFMDRS‐M scores evolution after DBS implantation according to the type of mutation. Missense mutations (n = 3, blue) and PTV (n = 22, orange). Compound heterozygote (p.Glu96Lys; p.Leu705Val), P21, was excluded from the graph (baseline and last BFMDRS‐M 3 and 1.5 points, respectively). [Color figure can be viewed at www.annalsofneurology.org]

Position of non‐responders in protein conformation is illustrated on Figure 3A, B. In patients with missense variants (compound heterozygote, P21 was not included), the disease onset appears to be lower (9.7 ± 2.9 years, n = 3 versus 18.6 ± 11.2 years, n = 22, *p* = 0.071) with higher BFMDRS‐M at baseline before DBS (61.2 ± 26.0, n = 3 vs 43.6 ± 20.4, n = 22, *p* = 0.225) (Fig 3C) than in those with PTV, but the results were not statistically significant. Statistically significant BFMDRS‐M improvement between baseline and last FU was observed only in PTV variants (*p* = 0.001) (Fig 3D). However, the interpretation of these results is limited by the sample size.

Statistically significant differences between non‐responders and responders were observed only for presence of fixed skeletal deformities (*r*_*φ*_ = −0,535, *p* = 0.006) and spasticity (*r*_*φ*_ = −0,479, *p* = 0.015). Although non‐responders had a younger age of onset (12.0 ± 8.4 vs 19.9 ± 11.1 years), younger age at DBS implantation (27.4 ± 17.3 vs 38.2 ± 13.1 years), shorter disease duration at DBS surgery (15.4 ± 11.5 vs 18.3 ± 9.1 years), and higher baseline BFMDRS‐M (51.6 ± 23.4 vs 41.3 ± 22.2 points), these results were not statistically significant. Based on binomial logistic regression for statistically significant factors, likelihood of being a non‐responder was 13‐fold higher for the presence of fixed skeletal deformities before DBS, and spasticity was not statistically significant anymore in this model. Detailed clinical characteristics of non‐responders are outlined in Supporting Information.

Considering BFMDRS‐D, 9/23 (39.1%) patients improved less than 25% at last FU compared to baseline (Table 2). A statistically significant predictor of BFMDRS‐D non‐responder status in regression analysis was not found.

### Pallidal Spiking Pattern for DYT‐VPS16

**FIGURE 4:**
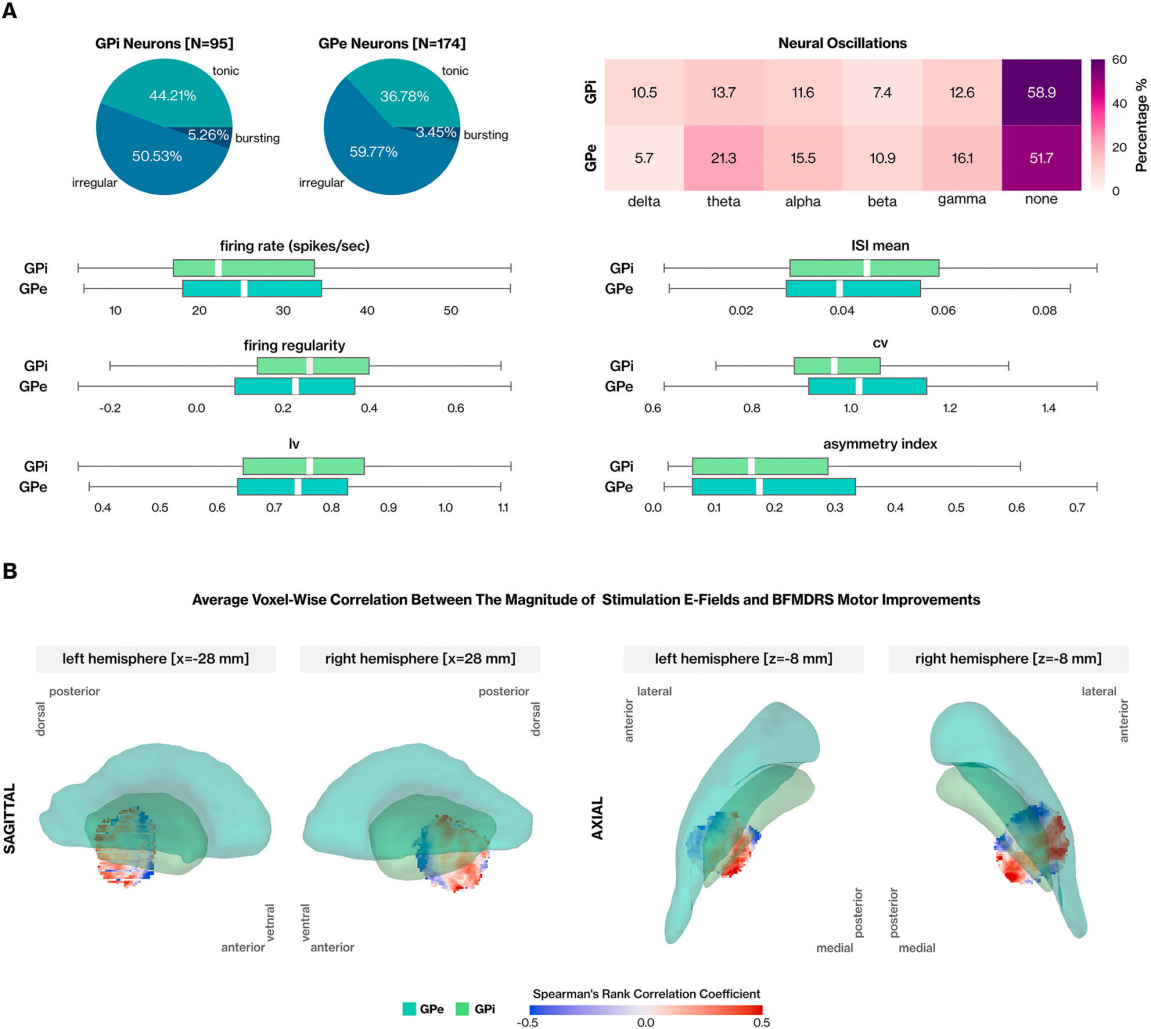
Comparative analysis of and globus pallidus internus (GPi) and globus pallidus externus (GPe) neural activity and deep brain stimulation (DBS) sweet and sour spot mapping for DYT‐*VPS16*. (A) The fraction of neurons exhibiting distinct spiking patterns in GPi and GPe is depicted in a pie chart, while a heatmap illustrates the proportion of neuron types displaying oscillatory behavior across various frequency bands. Box plots compare 6 selected neural features between the 2 nuclei. (B) Average voxel‐wise correlations between stimulation electric fields (E‐field) magnitudes and Burke‐Fahn‐Marsden Dystonia Rating Scale‐motor (BFMDRS‐M) score improvements are shown in sagittal (left) and axial (right) planes. The color‐coded reconstruction of GPi and GPe was performed using the Distal Atlas (with p > 0.5 thresholds for defining nuclei borders) via the Lead DBS v2.5 suite. [Color figure can be viewed at www.annalsofneurology.org]

The firing rates of GPi and GPe neurons were comparable, with 22.29 ± 16.82 (median ± interquartile range) and 25.36 ± 16.55 spikes/sec, respectively, (Dunn's test, *p* = 0.31) (Fig 4A). Spiking regularity metrics indicated similarities between the 2 nuclei, where firing regularity recorded as 0.26 ± 0.26 for GPi and 0.23 ± 0.28 for GPe (Dunn's test, *p* = 0.18) (see Fig 4A). The coefficient of variation (CV) was recorded as 0.97 ± 0.17 and 1.02 ± 0.24 for GPi and GPe. The majority of neurons (≥50%) exhibited irregular spiking in both nuclei. Contrarily, neural bursts were a rare phenomenon with only 5.26% and 3.45% of neurons presenting bursts. Approximately 45.7% of pallidal neurons presented significant oscillations in at least 1 frequency band, and the degree of neural oscillations remained comparable between GPi and GPe (Fisher exact test, *p* = 0.3) (see Fig 4A). Approximately 70% of pallidal oscillatory neurons exhibit significant oscillatory behavior in either the theta (4–8 Hz) range (40.65%) or the alpha (8–12 Hz) range (30.89%). Our findings further underscore the significance of pallidal low‐frequency oscillations regarding the dystonia pathophysiology, even at the single‐neuron level for monogenetic forms like DYT‐*VPS16*. Further detailed comparisons can be found in Table S2.

### Sweet and Sour Spots for DYT‐VPS16 Patients

We performed voxel‐wise correlation analysis between the stimulation E‐field magnitude and postoperative BFMDRS motor improvement across patients (n = 22) to generate sweet and sour spot mappings for DYT‐*VPS16*. To visualize the average voxel‐wise correlations in specific planes, correlation heatmaps were placed at the lateral‐most boundary (x = −28mm and 28mm for the left and right hemispheres, respectively) for the sagittal plane, and at the ventral‐most boundary (z = −8mm) of the globus pallidus for the axial plane (Fig 4B). At first glance, a noticeable gradient along the anteroposterior axis was observed, with sweet‐spot voxels predominantly located in the posterior region of the GPi, in contrast to sour‐spot voxels (see Fig 4B). Additionally, sour‐spot voxels are primarily located in the dorsolateral GPi, whereas stimulation of ventral segments is associated with better pallidal DBS outcomes (see Fig 4B). Individual GPi lead positions in axial, coronal, and sagittal planes for both sides, including responder status are summarized in Figure S1.

## Discussion

This is the largest cohort of patients with DYT‐*VPS16* reported to date, in which we report correlations between clinico‐demographic parameters, surgical factors, and genetic variants' spectrum and DBS outcomes, providing valuable data for DBS prognostication in this distinct clinico‐genetic syndrome. In 2 DYT‐*VPS16* patients (2/26), we analyze and report perioperative neurophysiological MER findings. In our cohort, 18 different variants were present, 4 of which were novel (c.1A>G, c.290 T>A, c.1189A>G, c.1204‐2A>G), not previously described in mutation databases (ClinVar) or MDSgene systematic literature review.^36^

*VPS16*‐related dystonia was previously clinically characterized by a craniocaudal gradient of dystonia with progressive generalization,^36^ which is consistent with findings seen also in our cohort. Most of our patients showed craniocervical or upper limb involvement with generalization in 23 of 26 cases at the time of DBS implantation. However, 5 of our patients (19.2%) reported initial lower limb involvement with further generalization, suggesting an atypical caudocranial course.

DYT‐*VPS16* usually presents as isolated dystonia, nevertheless, hyperkinetic comorbidities such as myoclonus^5^, ^7^, ^9^, ^13^ or choreathetosis^13^ have been mentioned previously. Four patients, 3 previously published (P13,^13^ P16,^13^ P23^7^) and 1 newly described (P21), with a combined myoclonus‐dystonia phenotype were present in our cohort (Table 2). P21 (compound heterozygote‐p.E96K; p.L705V) presenting with dystonia and myoclonus underwent VIM implantation and bilateral GPi‐DBS in a single surgery with excellent clinical benefit and no reported complications. High frequency stimulation of both VIM electrodes resulted in complete suppression of myoclonic head jerks, which had recurred after a microlesioning effect had dissolved 1 month after surgery. Additional bilateral GPi stimulation, which was programmed independently 2 months later, alleviated cervical dystonia that remained present despite VIM stimulation. Another patient, P14 (c.1204‐2A>G) underwent unilateral GPi (right) because of a complex phenotype of dystonia accompanied by psychosis and still improved 32.35% in BFMDRS‐M at last FU as compared to baseline.

Genetic background seems to be an important predictor of GPi‐DBS efficacy in patients with dystonia.^15^ Most of the available evidence in monogenic dystonia in this regard is, however, based on small numbers. Therefore, systematic reporting of DBS effects, both positive and negative, in monogenic dystonia is crucial to better understand its predictive value and support/assist decision‐making for DBS and patient information. Of 17 patients with *VPS16‐*related dystonia and implanted DBS reported to date (13 of them included in this study Table 1), 11 had significant improvement,^1^, ^6^, ^7^, ^9^, ^13^, ^37^ 1 had partial benefit,^1^ 2 patients were reported as “no use DBS”^1^ without clearly explaining whether it was ineffective or not, 2 patients had no reported efficacy yet,^8^ and recently 1 patient with dystonic tremor was successfully treated with VIM DBS^38^. Although these reports suggest a potential benefit of DBS also in DYT‐*VPS16* dystonia, our study supports and extends these data reporting on a DYT‐*VPS16* cohort of 26 patients with this rare monogenic disorder.

Overall, it appears that neuropsychiatric features (anxiety, emotional lability, and depression) are more prevalent than in some monogenic types of dystonia (eg, DYT‐*TOR1A* or even DYT‐*SGCE*).^36^ They also seem to be secondary in a proportion of patients and could be alleviated with effective treatment of dystonia. Although cognitive deterioration occurred in 2 patients in our cohort after DBS implantation, this finding should be interpreted with caution. In P5, this was based only on the treating neurologist's examination without formal neuropsychological testing (accentuated difficulties in decision making and following instructions, with cognitive difficulties were present already before surgery). In P7, this was based on formal neuropsychological testing, yet cognition was borderline normal at baseline, while meeting mild cognitive impairment criteria at FU. As we generally lack reported (especially longitudinal) neuropsychological data in *VPS16* dystonia, it is not clear whether this deterioration occurred because of stimulation side effects or a natural progression of the disease, which appeared to be rather mild in both cases.

DBS was efficacious in approximately three‐quarters of the patients for motor symptoms and in approximately half of the cohort for disability, despite some potential progression of the disease even under DBS. Less body parts were affected by dystonia in our cohort at the last FU as compared to baseline for all regions, except for pharyngeal dystonia, which was still present at the last FU in about one‐third of our patients (7/26, 26.9%) (see Fig 2A). This is in line with previous studies in generalized dystonia showing less benefit for the craniocervical region including swallowing and speech,^39^, ^40^, ^41^ but the efficacy of pallidal DBS for speech and laryngeal dystonia in our cohort seems to be higher as compared to DYT‐*KMT2B*.^42^ The recently described efficacy of thalamic DBS in a blinded, randomized controlled trial of adductor spasmodic dystonia^43^ and further tractography study^44^ support targeting of thalamic sensorimotor areas and involvement of the additional cerebellar circuit, not the pallidal one, in case of vocal cord motor activity.

As an observation related to the genetic background, most of the non‐responders (5/7) have variants situated between c.1188 and c.1814 of the gene, corresponding to amino acid residues p.396–605, a functionally uncharacterized region. In this region, there is only 1 responder (P14, c.1204‐2A>G) and even this patient responds to DBS just above the set limit for responsiveness, 32.4% in BFMDRS‐M at the last FU. Furthermore, in the region of amino acid residues 642–736, which seems to be necessary and sufficient for the interaction of VPS16 and VPS33A in the model of HOPS complex described to date,^45^ 5 of our patients were identified with 3 different variants (p.Arg647*, p.Gln714*, p.Lys724Glufs*44). All these patients were classified as high responders (Table 1, Fig 3A, and Table S1).

The mean overall significant improvement at the last FU compared to baseline for the whole group was 41.6% (26/26) and 34.8% (23/26) in BFMDRS‐M and BFMDRS‐D, respectively. When patients with a FU period of ≤1 year (P8, P9) were excluded to evaluate the long‐term effect of DBS, the mean improvement at the last FU was 41.3% and 33.4%, respectively. Significant improvements compared to baseline were also achieved for both mean BFMDRS‐M and BFMDRS‐D scores at the first FU, at 6 months FU for BFMDRS‐M and the highest improvement at 1‐year post‐DBS FU for both sub‐scores. However, these short‐term results are limited by the inconsistent subset of patients included in the evaluation because of data availability (see Fig 2B, C, Table S1).

Our study highlights the importance of DBS implantation before the onset of musculoskeletal deformities or of making the patient aware of the potentially insufficient effect of surgery once they are present as long as these are the only statistically significant negative predictors of BFMDRS‐M improvement in our cohort. Binomial logistic regression for statistically significant factors has shown that the likelihood of being a non‐responder to DBS is 13‐fold higher if the patient had fixed skeletal deformities before implantation. This relationship was not observed for the disability sub‐score.

Our sweet and sour spot estimation for DYT‐*VPS16* aligned with previous findings. Tisch et al^46^ suggested the more posterior placement of DBS leads resulting in better response to pallidal stimulation for generalized dystonia patients. The sweet‐spot region that we defined also aligns with the antidystonic sweet‐spot region (ventroposterior GPi) defined by Reich et al.^34^ A study focusing on the optimal stimulation target for DYT‐*TOR1A*, involving a similar number of patients, also suggested that the optimal region is partially contained within the posterior section of the GPi for this monogenic form of dystonia.^47^ Horn et al^33^ proposed that optimal stimulation sites within the pallidum correspond to somatotopic pallidal regions, where focused ventroposterior stimulation is ideal for cervical dystonia, whereas a more widespread mapping is preferred for generalized dystonia. In this regard, we did not conduct an analysis considering the clinical phenotype of our patients.

There were no other statistically significant differences between responders and non‐responders, but overall non‐responders had a younger age of onset, younger age at DBS implantation, shorter disease duration at DBS surgery, and higher baseline BFMDRS‐M compared to responders. This contrasts with the worse motor outcome with longer dystonia duration and older age at dystonia onset in patients with DYT‐*TOR1A* or longer dystonia duration in DYT/PARK‐*TAF1*.^14^ However, a better motor outcome with older age at onset was also associated with DYT*‐SGCE*.^14^ Although the implantation of DBS at a younger age in patients with *VPS16* dystonia seems to lead to worse outcomes, these results should be interpreted with caution. Age alone may not be a decisive parameter for DBS surgery, but in the context of other findings, such as significant motor deficits leading to early fixed deformities and spasticity, it could help to set the realistic expectations from procedure to the patients. Although non‐responders in our study tended to be younger, with faster disease progression and worse disease severity before surgery, it seems that early DBS implantation, before these complications occur is a more important factor of long‐term DBS efficacy, rather than the more malignant phenotype of the disease in this subgroup of patients. Early DBS implantation should, therefore, be generally considered as an important factor of long‐term DBS success across the spectrum of isolated monogenic dystonias.

The neural activity of the GPi and GPe was comparable, with both nuclei demonstrating a high degree of spiking irregularities and infrequent bursts. This finding is partially consistent with the only available published single unit activities for DYT‐*VPS16*, which reported a prominent pallidal spiking irregularity (56.8%) alongside moderate burstiness (20.3%).^35^ The observed discrepancies may be attributed to differences in anesthetic administration protocols.

We acknowledge limitations to our study: the relatively small number of patients enrolled because of rarity of the disorder, the retrospective design as well as involvement of different European centers with variable FU protocols and rather variable stimulation parameters settings could influence the outcomes of this study. Furthermore, BFMDRS was the only parameter consistently used to assess outcomes. Other important indicators were not available to address the full complexity of the disorder and of related impairments and consequences. This raises a strong need for a minimal standard recommendation/guideline of FU and reporting in DBS studies for dystonia, which would significantly increase harmonization of data across different centers and cohorts, eventually leading to improved interpretability of DBS reporting studies in dystonia. We believe that this will lead also to better prediction of outcome in cases with genetic dystonia that have not yet been implanted.

In conclusion, bilateral pallidal DBS appears to be a safe and effective treatment for dystonic features in majority of patients with DYT‐*VPS16*. Although DBS seems to be generally well effective for all body regions, except pharyngeal dystonia, it should be considered early before spasticity and fixed skeletal deformities occur, because these are statistically significant predictors of worse DBS outcomes once they are present. Patients with medically refractory *VPS16‐*related dystonia should be informed about this surgical alternative.

## AUTHOR CONTRIBUTIONS

**Tatiana Svorenova:** Conceptualization; formal analysis; investigation; methodology; project administration; resources; validation; visualization; writing – original draft; writing – review and editing. **Luigi M. Romito:** Conceptualization; data curation; formal analysis; investigation; methodology; resources; visualization; writing – original draft; writing – review and editing. **Ahmet Kaymak:** Conceptualization; data curation; formal analysis; investigation; methodology; software; validation; visualization; writing – original draft; writing – review and editing. **Eoin Mulroy:** Investigation; resources; writing – review and editing. **Laura Cif:** Investigation; resources; validation; writing – review and editing. **Elena Moro:** Investigation; resources; writing – review and editing. **Kirsten E. Zeuner:** Investigation; resources; writing – review and editing. **Simone Zittel:** Investigation; resources; writing – review and editing. **Jan Niklas Petry‐Schmelzer:** Formal analysis; investigation; resources; validation; writing – review and editing. **Doreen Gruber:** Investigation; resources; writing – review and editing. **Liesanne Centen:** Investigation; resources; writing – review and editing. **Alberto Albanese:** Investigation; resources; writing – review and editing. **Miriama Ostrozovicova:** Investigation; resources; writing – review and editing. **Vladimir Han:** Investigation; resources; writing – review and editing. **Veronika Magocova:** Investigation; resources; writing – review and editing. **Kamil Knorovsky:** Investigation; resources; writing – review and editing. **Aurelia Kollova:** Investigation; resources; writing – review and editing. **Barbara Garavaglia:** Investigation; resources; writing – review and editing. **Nico Golfrè‐Andreasi:** Investigation; resources; writing – review and editing. **Chiara Reale:** Investigation; resources; writing – review and editing. **Alberto Mazzoni:** Investigation; resources; writing – review and editing. **Giovanna Zorzi:** Investigation; resources; writing – review and editing. **Roberto Eleopra:** Investigation; resources; writing – review and editing. **Vincenzo Levi:** Investigation; resources; writing – review and editing. **Thomas Foltynie:** Investigation; resources; writing – review and editing. **Patricia Limousin:** Investigation; resources; writing – review and editing. **Harith Akram:** Investigation; resources; writing – review and editing. **Ludvic Zrinzo:** Investigation; resources; writing – review and editing. **Francesca Magrinelli:** Investigation; resources; writing – review and editing. **David Murphy:** Investigation; resources; writing – review and editing. **Henry Houlden:** Investigation; resources; writing – review and editing. **Manju A. Kurian:** Investigation; resources; writing – review and editing. **Claudio Baiata:** Investigation; resources; writing – review and editing. **Steffen Paschen:** Investigation; resources; writing – review and editing. **Katja Lohmann:** Investigation; resources; writing – review and editing. **Jens Volkmann:** Investigation; resources; writing – review and editing. **Wolfgang Hamel:** Investigation; resources; writing – review and editing. **Michael T. Barbe:** Investigation; resources; writing – review and editing. **Martje E. van Egmond:** Investigation; resources; writing – review and editing. **MAJ Tijssen:** Investigation; resources; writing – review and editing. **Lubos Ambro:** Formal analysis; investigation; visualization; writing – review and editing. **Veronika Jurkova:** Formal analysis; methodology; validation; writing – review and editing. **Robert Jech:** Investigation; resources; writing – review and editing. **Petra Havrankova:** Investigation; resources; writing – review and editing. **Juliane Winkelmann:** Investigation; resources; writing – review and editing. **Michael Zech:** Formal analysis; investigation; resources; validation; writing – review and editing. **Matej Skorvanek:** Conceptualization; data curation; formal analysis; funding acquisition; investigation; methodology; project administration; resources; supervision; validation; writing – original draft; writing – review and editing.

## Potential Conflicts of Interest

M.T.B. received speaker's honoraria from DBS manufacturers Medtronic, Boston Scientific, Abbott (formerly St. Jude), as well as advisory honoraria and research funding from Medtronic (ODIS, OPEL, BeAble) and Boston Scientific. M.S. received speaker's honoraria and consultation fees from Medtronic, the company that produces DBS. The other authors report no competing interests.

## Supporting information


**Data S1.** Supporting Information.

Figure S1.

Table S1.

Table S2.

## Data Availability

The complete code for MER analysis, DBS sweet and sour spot mapping analysis, and plotting is available online on the GitHub repository: https://github.com/ahmetofficial/DYT-VPS16-Analyses. The data that support the findings of this study are available in Supporting information and on reasonable request from the corresponding authors.
