# Supplementary material for: Deep Brain Stimulation for VPS16 ‐Related Dystonia: A Multicenter Study
Source: Ann Neurol. 2025 Jun 20;98(4):711–25. doi: 10.1002/ana.27290 (PMC12542321; doi:10.1002/ana.27290)

**Supplementary materials for:**

**Deep brain stimulation for *VPS16*-related dystonia: A multicenter study**

Tatiana Svorenova^1,2^**^†^**; Luigi M Romito^3^**^†^**; Ahmet Kaymak^4^**^†^**; Eoin Mulroy^5^; Laura Cif^6,7^; Elena Moro^8^, Kirsten E. Zeuner^9^; Simone Zittel^10^; Jan Niklas Petry-Schmelzer^11^; Doreen Gruber^12^; Liesanne Centen^13,14^; Alberto Albanese^15^; Miriama Ostrozovicova,^1,2,16^; Vladimir Han^1,2^; Veronika Magocova^17,18^; Kamil Knorovsky^17,18^; Aurelia Kollova^17,18^; Barbara Garavaglia^19^; Nico Golfrè-Andreasi^3^; Chiara Reale^19^; Alberto Mazzoni^4^; Giovanna Zorzi^20^; Roberto Eleopra^3^; Vincenzo Levi^21^; Thomas Foltynie^5^; Patricia Limousin^5^; Harith Akram^5^; Ludvic Zrinzo^5^; Francesca Magrinelli^22^; David Murphy^22^; Henry Houlden^23^; Manju A Kurian^24,25^; Claudio Baiata^26^; Steffen Paschen^9^; Katja Lohmann^27^; Jens Volkmann^28^; Wolfgang Hamel^29^; Michael T. Barbe^11^; Martje E van Egmond^13,14^; MAJ Tijssen^13,14^; Lubos Ambro^30^; Veronika Jurkova^31^; Robert Jech^32^; Petra Havrankova^32^; Juliane Winkelmann^33,34,35^; Michael Zech^33,34,36^; Matej Skorvanek^1,2^

^†^These authors contributed equally to this work.

**METHODS**

***Recruitment and ethical approval***

The list of centres from Europe, Americas, Asia and Australia contacted for the availability of patients with DYT-*VPS16* dystonia and implanted DBS:

Europe

1. P.J. Safarik University and University Hospital of L. Pasteur, Kosice, Slovak Republic
2. Fondazione IRCCS Istituto Neurologico Carlo Besta, Milan, Italy
3. IRCCS Fondazione Mondino, Pavia, Italy
4. University of Padua, Padova, Italy
5. University of Salerno, Fisciano, Italy
6. Hospital Ruber Internacional, Madrid, Spain
7. University of Navarra, Madrid, Spain
8. University of Barcelona, Spain
9. Sant Pau Hospital, Barcelona, Spain
10. North Lisbon University Hospital Center, Lisbon, Portugal
11. Innsbruck University Hospital, Innsbruck, Austria
12. Charité Universitätsmedizin Berlin, Berlin, Germany
13. University of Würzburg, Würzburg, Germany
14. Universitätsklinikum Schleswig-Holstein, Campus Kiel, Kiel, Germany
15. University Medical Center Hamburg-Eppendorf, Hamburg, Germany
16. Movement Disorders Hospital, Beelitz-Heilstätten, Germany
17. University of Cologne, Cologne, Germany
18. University of Luebeck, Luebeck, Germany
19. Hannover Medical School Hospital, Hannover, Germany
20. Universitätsklinikum Giessen und Marburg, Standort Marburg, Germany
21. Tübingen University Hospital, Tübingen, Germany
22. Great Ormond Street Hospital for Children, London, United Kingdom
23. UCL Queen Square Institute of Neurology, London, United Kingdom
24. University Medical Center Groningen, University of Groningen, the Netherlands
25. Leiden University Medical Centre, Leiden, Netherlands
26. University Hospital Montpellier Montpellier, France
27. CHU of Grenoble, Grenoble Institute of Neurosciences, Grenoble, France
28. Pitié-Salpêtrière Hospital, Sorbonne University, Paris, France
29. University of Lille, Lille, France

Australia

1. Faculty of Medicine and Health, University of Sydney, Australia

Americas

1. Toronto Western Hospital, Toronto, Canada
2. University of Cincinnati Gardner Neuroscience Institute, Ohio, United States
3. University Of Florida, Gainesville, Florida, United States
4. Rady Children's Hospital San Diego, California, United States
5. UT Southwestern Medical Center in Dallas, Texas, United States

Asia

1. Juntendo University in Tokyo, Tokyo, Japan
2. Seoul National University Hospital, Seoul, South Korea

***DBS lead localizations and e-field modelling***

We localized DBS electrodes for the patients in our cohort using the advanced processing pipeline available in Lead-DBS v2.5^1^. Briefly, postoperative CT or MRI scans were linearly coregistered to preoperative T1-weighted images using Advanced Normalization Tools (ANTs)^2^. This process included careful inspection and manual refinement applied if needed. DBS electrode localizations were adjusted for brain shifts in postoperative scans by applying a refined affine transform restricted to a subcortical area of interest, as implemented in the brain-shift correction module of Lead-DBS^3^. Both pre- and postoperative scans were spatially normalized to the ICBM 2009b NLIN asymmetric (MNI) template space^4^ using the Symmetric Normalization (SyN) algorithm^5^ in ANTs. The normalization process involved five stages: two initial linear registration steps (rigid and affine transformations), a whole-brain nonlinear SyN registration, and two subsequent nonlinear SyN registrations focused specifically on the subcortical region of interest, guided by predefined subcortical masks^6^.

For electric fields (E-fields) estimation, we followed the methodology presented previously.^7^ E-fields were estimated in native space based on the long-term DBS settings applied, using an adaptation of the SimBio/FieldTrip pipeline^8^ implemented in Lead-DBS. The finite element method was employed to solve the static formulation of the Laplace equation on a discretized domain represented by a tetrahedral four-compartment mesh, which included grey matter, white matter, metal, and insulating electrode parts. The resulting E-fields were transformed into MNI space using the refined normalization warpfields, ensuring consistent spatial alignment for all analyses.

***DBS sweet and sour spot mapping***

To examine the impact of stimulation intensity and position on GPi-DBS responsiveness in DYT-*VPS16* patients, we applied a voxel-wise correlation analysis.^7,9^ Specifically, E-fields were modelled in 22 patients (3 patients were excluded from this analysis due to low image quality, **Supplementary Table 1**) based on active contacts and stimulation parameters, analysed separately for the right and left hemispheres. Points within E-field with vector magnitude below 150 V/m, the threshold assumed sufficient to activate axons^10^, were filtered out. We compiled the remaining E-field points with varying vector magnitudes for each 0.25x0.25x0.25 mm voxel in MNI space and calculated an average E-field vector magnitude per voxel. For robust voxel-based correlation estimation with Spearman´s rho test, we required each selected voxel (in either hemisphere) to be covered by at least 30%^7^ of patients (N≥7), otherwise these voxels were excluded from further analyses. For each voxel, we measured Spearman’s correlation between the E-field vector magnitude values and the percentual BFMDRS-M improvement following the GPi-DBS surgery across patients.

To identify and visualize sweet and sour spot regions based on voxel-wise correlation scores, we processed voxel data using a combination of statistical filtering and 3D visualization techniques for each hemisphere separately. We calculated the average correlation within a specific plane (axial, coronal, or sagittal) by aggregating the mean correlation coefficients across voxels sharing the same position on the selected plane. For instance, voxels with identical borders in the x and z axes were grouped in the axial plane. This method allowed us to visualize the spatial distribution of correlation values in a more interpretable manner, highlighting regions within the plane that exhibit the strongest positive or negative correlations. By aggregating data in this way, we could identify "sweet spots," where stimulation is most beneficial, and "sour spots," where it may have adverse effects. This approach enabled a clear delineation of sweet and sour spot regions, offering valuable insights into the anatomical-functional relationships that underpin the effects of GPi-DBS in DYT-*VPS16* cases.

***MER data analysis***

We examined intraoperative microelectrode recordings (MER) from five trajectories acquired during pallidal DBS surgery under propofol anesthesia in two patients (P8 and P9) to characterize the neural activity of the internal and external segments of the globus pallidus (GPi and GPe, respectively) for DYT-*VPS16*. The reconstruction of nuclei and recording depths are performed using the Distal Atlas^11^ in Montreal Neurological Institute space (p>0.5 thresholds for definitions of nuclei borders) by Lead DBS^1^ v2.5 and operating room notes. From raw recordings, we isolated 95 and 174 stable single-unit activities (SUA) from GPi and GPe, respectively. The methodology for spike sorting and neural feature measurements has been described in previous work.^12^

**RESULTS**

***DBS insertion and outcomes***

Non-responders included the patient with the shortest FU (2 months, P8), who had his battery discharged for 1 month and underwent DBS explantation 11 months after DBS surgery because of subjective ineffectiveness; but also, the patient with the longest FU (216 months, P10) in our cohort, who did not improve at all after DBS, unlike other non-responders who improved at least partially or temporarily. All non-responders had isolated generalised dystonia at baseline with onset in the extremities, except one non-responder, P12, who initially presented with laryngeal dystonia. Five of non-responders had at least one neuropsychiatric comorbidity. One non-responder, P11, had a mild facial nerve deficit as a complication of surgery.

**DISCUSSION**

Compared to other monogenic isolated dystonias, the mean age of onset of *VPS16*-related dystonia in our cohort was higher (17.8 years) than in DYT-*KMT2B*^13^, DYT-*TOR1A*^14^ or DYT-*THAP1*^14^*,* but lower than in DYT-*GNAL* dystonia^15^. This is consistent with the early, adolescent onset of *VPS16*-related dystonia as described in a systematic literature review based on previously published patients with a median age at onset of 14 years^16^. Nevertheless, five of our patients (19.2%) experienced their first dystonic symptoms at an age of >21 years.

**REFERENCES**

1. Horn A, Li N, Dembek TA, et al. Lead-DBS v2: Towards a comprehensive pipeline for deep brain stimulation imaging. *Neuroimage* 2019;184:293-316;DOI:10.1016/j.neuroimage.2018.08.068
2. Avants BB, Tustison NJ, Song G, et al. A reproducible evaluation of ANTs similarity metric performance in brain image registration. *NeuroImage* 2011;54(3):2033-2044;DOI:10.1016/j.neuroimage.2010.09.025
3. Horn A, Kühn AA. Lead-DBS: A toolbox for deep brain stimulation electrode localizations and visualizations. *NeuroImage* 2015;107:127-135;DOI:10.1016/j.neuroimage.2014.12.002
4. Fonov V, Evans AC, Botteron K, et al. Unbiased average age-appropriate atlases for pediatric studies. *NeuroImage* 2011;54(1):313-327;DOI:10.1016/j.neuroimage.2010.07.033
5. Avants BB, Epstein CL, Grossman M, et al. Symmetric diffeomorphic image registration with cross-correlation: evaluating automated labeling of elderly and neurodegenerative brain. *Med Image Anal* 2008;12(1):26-41;DOI:10.1016/j.media.2007.06.004
6. Schönecker T, Kupsch A, Kühn AA, et al. Automated Optimization of Subcortical Cerebral MR Imaging−Atlas Coregistration for Improved Postoperative Electrode Localization in Deep Brain Stimulation. *AJNR Am J Neuroradiol* 2009;30(10):1914-1921;DOI:10.3174/ajnr.A1741
7. Horn, A., Reich, M.M., Ewert, S. et al. Optimal deep brain stimulation sites and networks for cervical vs. generalized dystonia. *Proc Natl Acad Sci USA* 2022; 119(14); e2114985119;DOI:10.1073/pnas.2114985119
8. Vorwerk J, Oostenveld R, Piastra MC, et al. The FieldTrip-SimBio pipeline for EEG forward solutions. *BioMed Eng OnLine* 2018;17(1):37;DOI:10.1186/s12938-018-0463-y
9. Reich MM, Horn A, Lange F, et al. Probabilistic mapping of the antidystonic effect of pallidal neurostimulation: a multicentre imaging study. *Brain* 2019;142(5):1386-1398;DOI:10.1093/brain/awz046
10. Astrom M, Diczfalusy E, Martens H, et al. Relationship between neural activation and electric field distribution during deep brain stimulation. *IEEE Trans Biomed Eng* 2015;62(2):664-672;DOI:10.1109/TBME.2014.2363494
11. Ewert S, Plettig P, Li N, et al. Toward defining deep brain stimulation targets in MNI space: A subcortical atlas based on multimodal MRI, histology and structural connectivity. *Neuroimage* 2018;170:271-282;DOI:10.1016/j.neuroimage.2017.05.015
12. Kaymak A, Vissani M, Lenge M, et al. Patterns of Neural Activity and Clinical Outcomes in a Juvenile Huntington’s Disease Patient Undergoing Deep Brain Stimulation of the Subthalamic Nucleus*. Deep Brain Stimul* 2023;S2949669123000027;DOI:10.1016/j.jdbs.2023.03.001
13. Zech M, Boesch S, Maier EM, et al. Haploinsufficiency of KMT2B, Encoding the Lysine-Specific Histone Methyltransferase 2B, Results in Early-Onset Generalized Dystonia. *Am J Hum Genet* 2016;99(6):1377-1387;DOI:10.1016/j.ajhg.2016.10.010
14. Ozelius LJ, Bressman SB. Genetic and clinical features of primary torsion dystonia. *Neurobiol Dis* 2011;42(2):127-135;DOI:10.1016/j.nbd.2010.12.012
15. Fuchs T, Saunders-Pullman R, Masuho I, et al. Mutations in GNAL cause primary torsion dystonia. *Nat Genet* 2013;45(1):88-92;DOI:10.1038/ng.2496
16. Thomsen M, Lange LM, Klein C, et al. MDSGene: Extending the List of Isolated Dystonia Genes by VPS16, EIF2AK2, and AOPEP. *Mov Disord* 2023;38(3):507-508;DOI:10.1002/mds.29327

**FIGURES (COLOR BLIND FRIENDLY VERSION)**

**Figure 2 BFMDRS sub-scores evolution and dystonia distribution before and after DBS**.

**(A)** Distribution of dystonia of the cohort (N=26) before DBS at baseline and at the last follow-up, including last follow-ups <12 months after DBS (mean 102.5±57.3 months). Numbers in columns represent the number of affected patients. **(B)** Mean BFMDRS-M at baseline (N=26), 3 months (N=8), 6 months (N=6), 12 months (N=16) and at the last follow-up > 12 months after surgery (mean 110.4±52.0 months, N=24) **(C)** Mean BFMDRS-D at baseline (N=23), 3 months (N=8), 6 months (N=6), 12 months (N=15) and at the last follow-up > 12 months after surgery (mean 109.7±55.1 months, N=21) **(D)** Individual BFMDRS-M sub-scores evolution after DBS implantation (N=26) with following color-coding: purple for high responders (>50%), pink for responders (25-50%), brown for non-responders (<25% improvement in BFMDRS-M at the last follow-up compared to baseline). **(E)** Individual BFMDRS-D sub-scores evolution after DBS implantation (N=23) with color-coding based on improvement in BFMDRS-M.


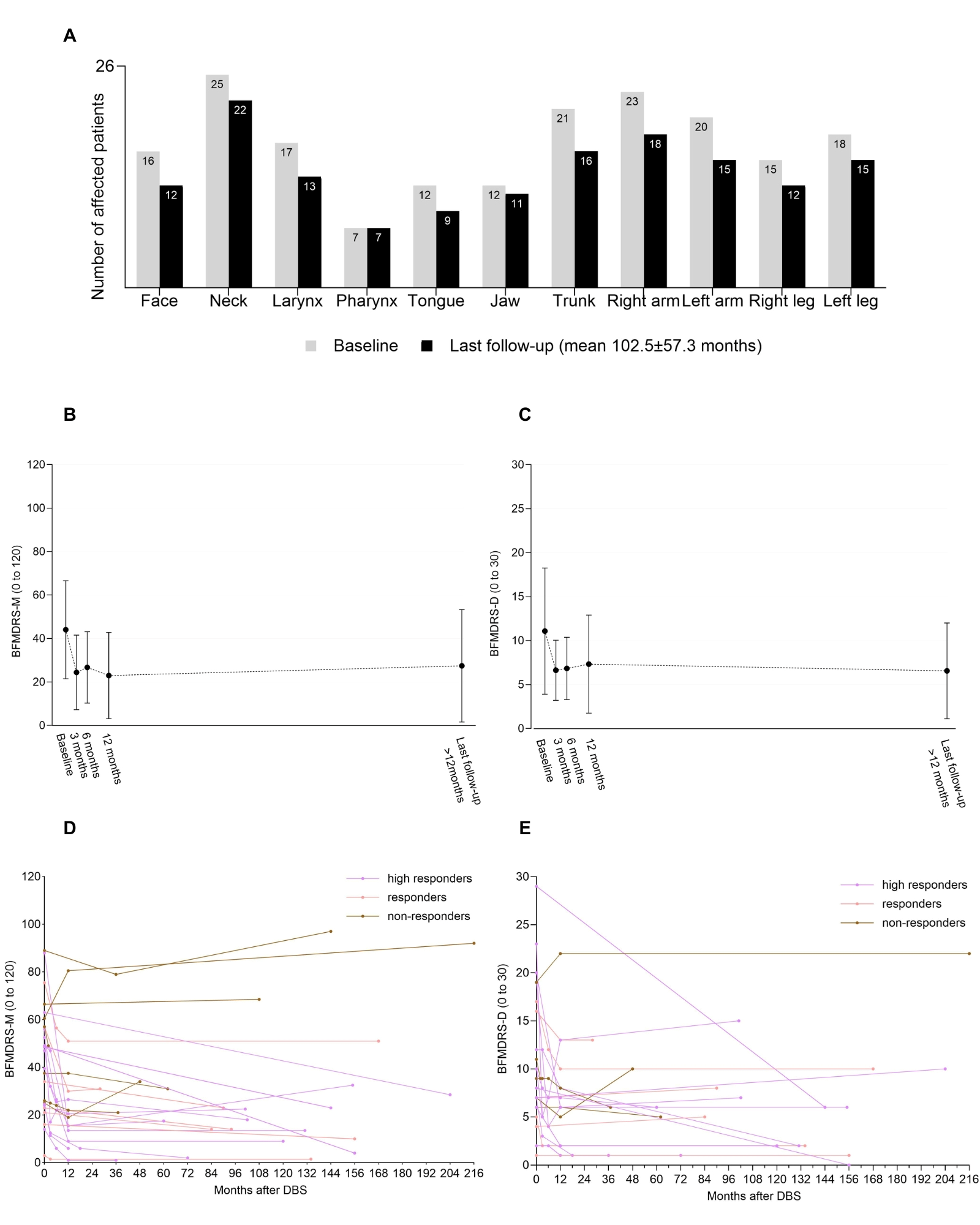


**Figure 3 Relationship between genotype, protein position and response to DBS in terms of dystonia severity.**

**(A)** Visualization of the mutations of patients with last follow-up >12 months after DBS analysed in this study mapped onto the AlphaFold model of the human VPS16 protein (orange ribbon, AlphaFold identifier: AF-Q9H269-F1). The model is overlaid with the crystal structure of a short fragment of human VPS16 (residues 642-736) in complex with human VPS33 (PDB ID: 4BX9). Target amino acid residues on VPS16 are represented as spheres, with following color-coding: purple for high responders (>50%), pink for responders (25-50%), brown for non-responders (<25% improvement in BFMDRS-M at the last follow-up compared to baseline) and grey for mixed responses to DBS. The VPS33 protein is displayed as a pale blue surface, highlighting its major interaction interface with VPS16 in the human HOPS complex. Missense mutations that were further analysed through bioinformatics predictions are labelled in bold, with the mutated amino acid in parentheses. Compound heterozygote (*), P21, was responder to DBS with 50% improvement in BFMDRS-M. Start-loss mutation (c.1A>G mixed responses) and splice-site mutations (c.1204-2A>G responder; c.1204-2A>G non-responder) analysed in our cohort are not related to the position on the protein. **(B)** Detailed view of the β-sheet-rich globular domain of human VPS16, showing mutations at positions Lys 82, Glu 96, Leu 97, Leu 155 and Arg 187. This close-up provides clarity on the spatial positions of these mutations. **(C)** Relationship between type of mutation and BFMDRS-M score at baseline before DBS. Missense mutations (N=3, blue) and protein truncating variants (PTV, N=22, orange). Compound heterozygote (p.Glu96Lys; p.Leu705Val), P21, was excluded from the graph (baseline BFMDRS-M 3 points). **(D)** Dystonia BFMDRS-M scores evolution after DBS implantation according to the type of mutation. Missense mutations (N=3, blue) and protein truncating variants (PTV, N=22, orange). Compound heterozygote (p.Glu96Lys; p.Leu705Val), P21, was excluded from the graph (baseline and last BFMDRS-M 3 and 1.5 points, respectively).


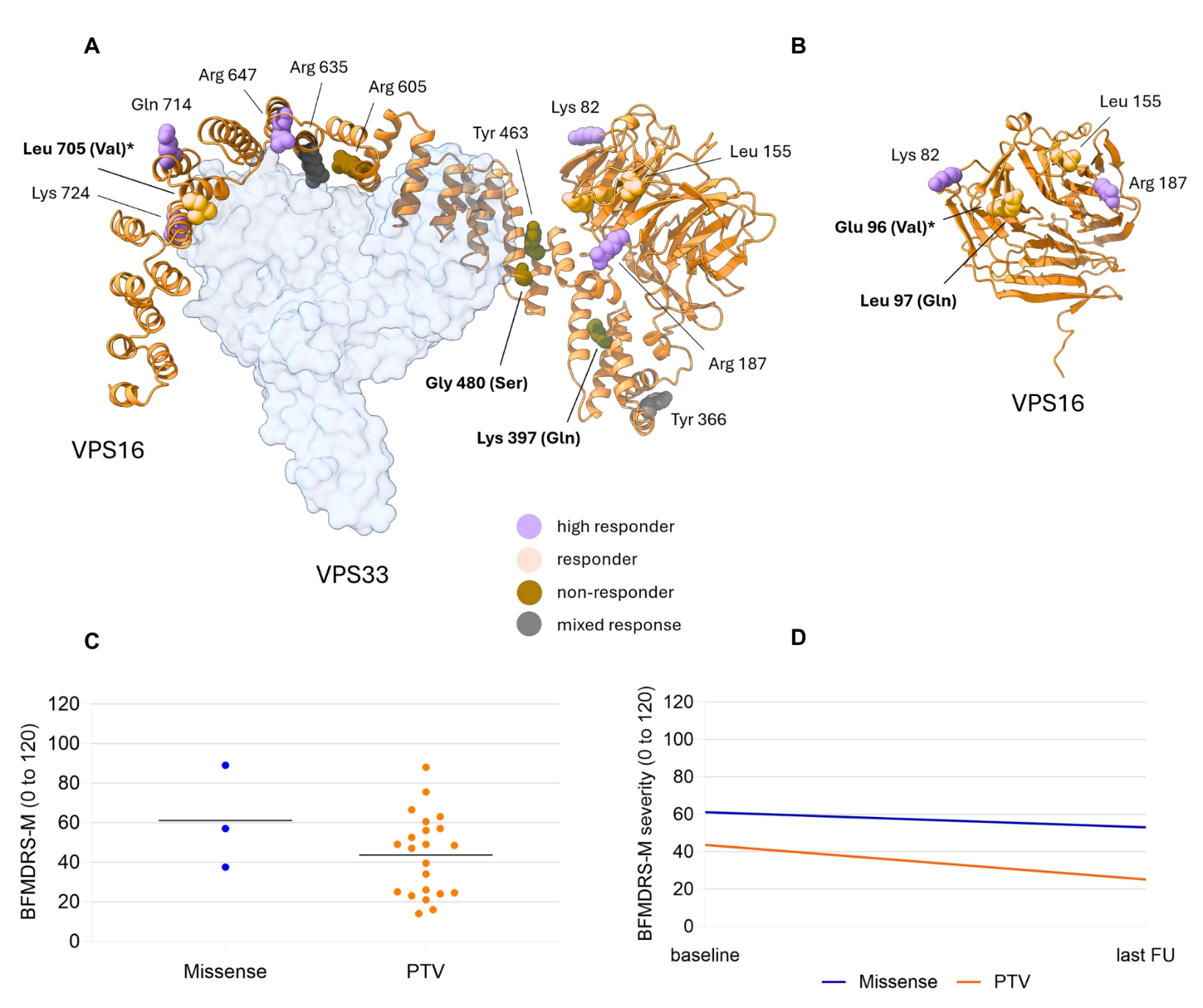

Supplement: Supplementary file 1 — Data S1. Supporting Information. [file ANA-98-711-s001.docx]
