# Supplementary material for: Deep Brain Stimulation for VPS16 ‐Related Dystonia: A Multicenter Study
Source: Ann Neurol. 2025 Jun 20;98(4):711–25. doi: 10.1002/ana.27290 (PMC12542321; doi:10.1002/ana.27290)

**Supplementary Figure 1 Neuroanatomical mapping of DBS lead positions in comparison with DBS outcomes.**

Visualisation of electrode positions in axial, coronal and sagittal planes for both sides at different depth levels. The reconstruction of GPi, GPe, and STN was performed using the Distal Atlas in Montreal Neurological Institute space (with p>0.5 thresholds for defining nuclei borders) via the Lead DBS v2.3 suite. The stimulation centers for three patient groups are color-coded: red for non-responders (<25% improvement in BFMDRS-Motor subscale at the last follow-up compared to baseline), blue for responders (25-50%), green for high responders (>50%) to DBS.


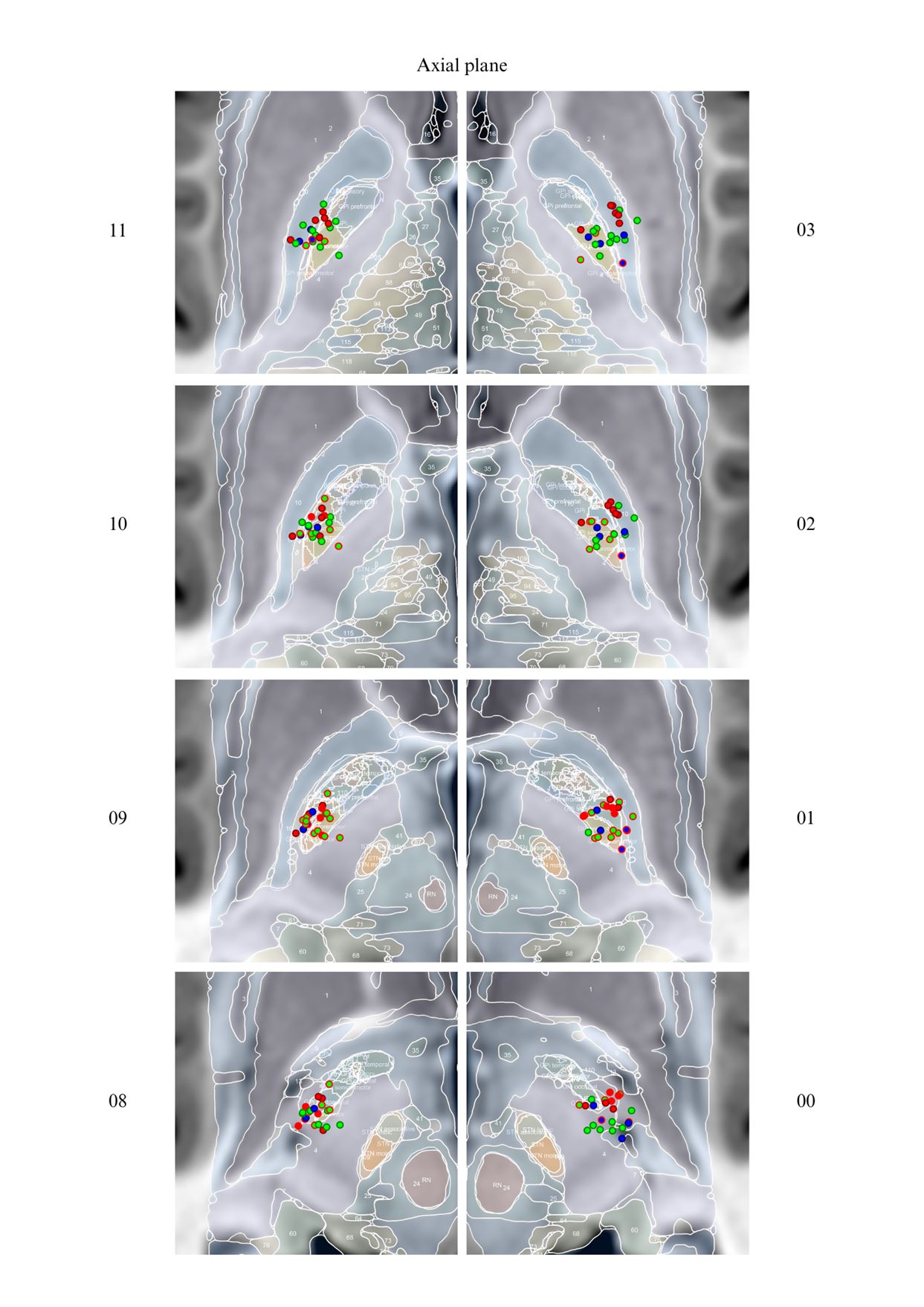


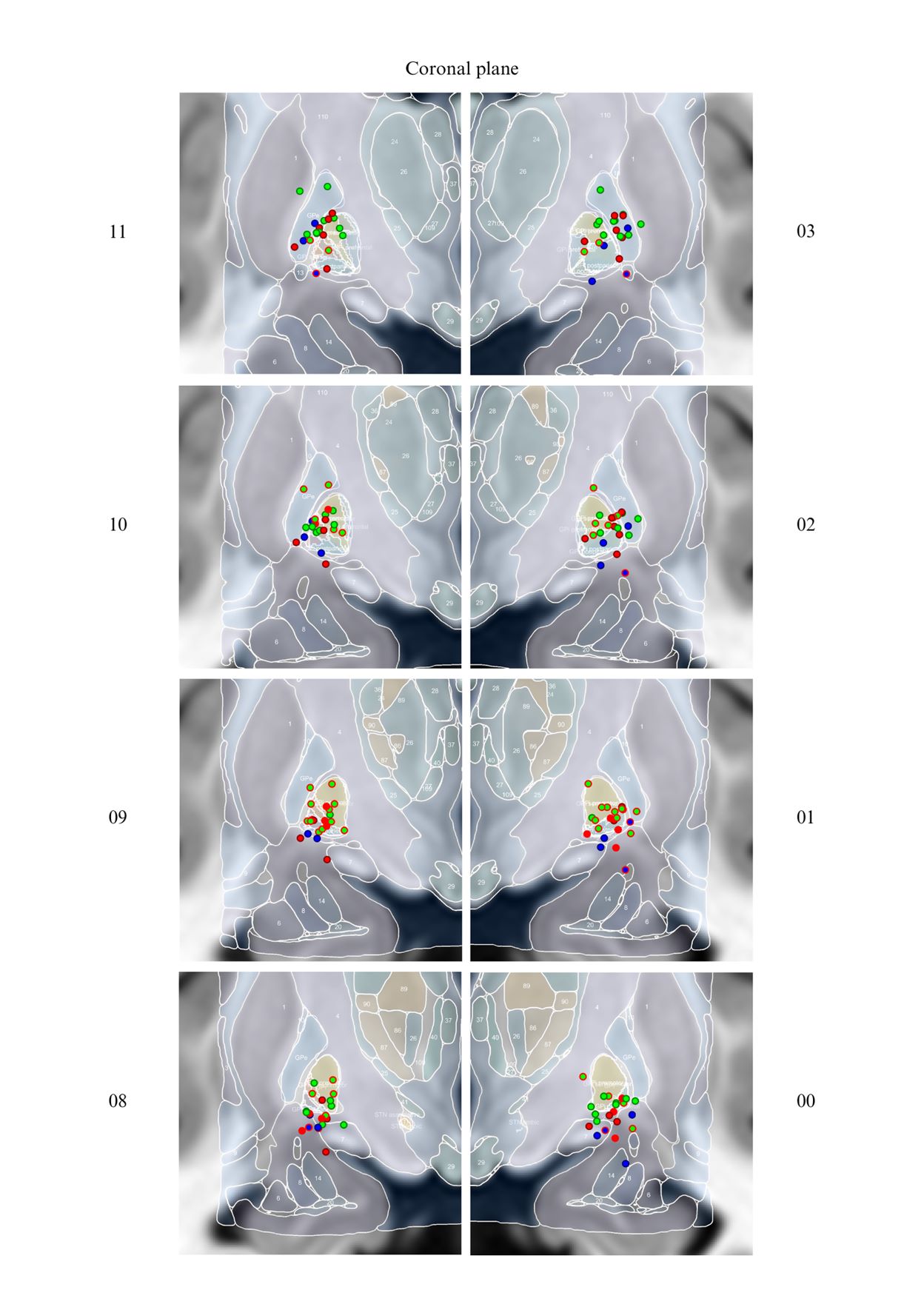

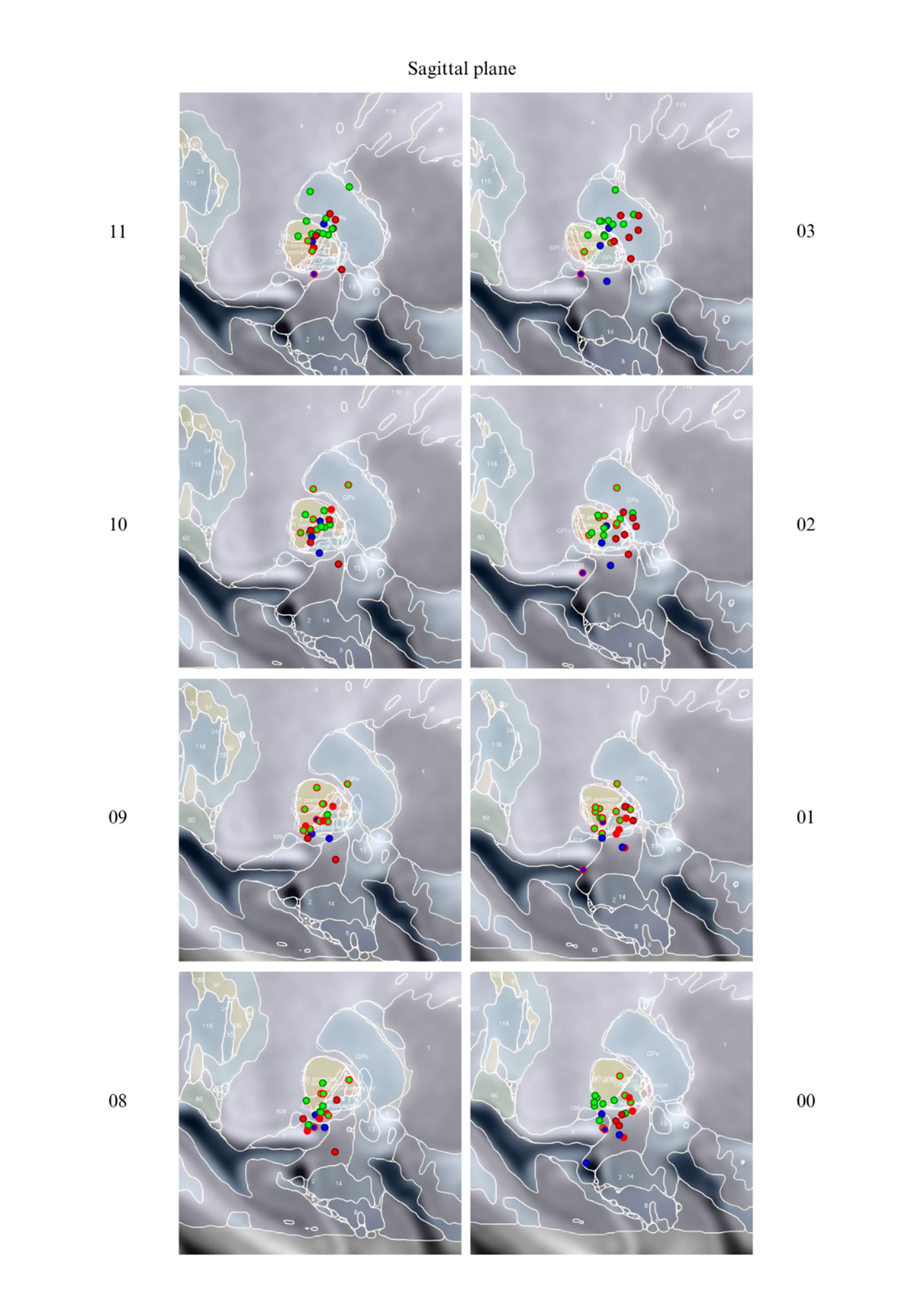

Supplement: Supplementary file 2 — Figure S1. [file ANA-98-711-s002.docx]
