# Supplementary material for: Deep Brain Stimulation for VPS16 ‐Related Dystonia: A Multicenter Study
Source: Ann Neurol. 2025 Jun 20;98(4):711–25. doi: 10.1002/ana.27290 (PMC12542321; doi:10.1002/ana.27290)
Supplement: Supplementary file 4 — Table S2. [file ANA-98-711-s003.docx]

| Neural Features | GPi | GPe | *p*-value |
| --- | --- | --- | --- |
| continuous | | | |
| firing rate (spikes/sec) | 22.29 ± 16.82 | 25.36 ± 16.55 | 0.31 |
| firing regularity | 0.26 ± 0.26 | 0.23 ± 0.28 | 0.18 |
| cv | 0.97 ± 0.17 | 1.02 ± 0.24 | 0.10 |
| lv | 0.76 ± 0.21 | 0.74 ± 0.19 | 0.29 |
| ISI mean | 0.04 ± 0.03 | 0.04 ± 0.03 | 0.31 |
| ISI std | 0.04 ± 0.03 | 0.04 ± 0.03 | 0.55 |
| ISI skewness | 1.75 ± 0.23 | 1.78 ± 0.25 | 0.18 |
| ISI correlation coefficient | 0.01 ± 0.12 | 0.03 ± 0.14 | 0.20 |
| asymmetry index | 0.16 ± 0.22 | 0.18 ± 0.27 | 0.58 |
| theta [4-8 Hz] oscillation frequency [Hz] | 5.49 ± 1.1 | 5.13 ± 1.83 | 0.81 |
| alpha [8-12 Hz] oscillation frequency [Hz] | 8.79 ± 2.2 | 8.79 ± 2.2 | 0.96 |
| beta [12-30 Hz] oscillation frequency [Hz] | 14.65 ± 9.52 | 14.74 ± 10.25 | 0.99 |
| gamma [30-100 Hz] oscillation frequency [Hz] | 44.68 ± 26.37 | 41.02 ± 24.9 | 0.92 |
| binary | | | |
| bursting neurons (%) | 5.26 | 3.45 | 0.53 |
| tonic neurons (%) | 44.21 | 36.78 | 0.24 |
| irregular neurons (%) | 50.53 | 59.77 | 0.16 |
| delta band [1-4 Hz] oscillatory neurons (%) | 10.53 | 5.75 | 0.22 |
| theta band [4-8 Hz] oscillatory neurons (%) | 13.68 | 21.26 | 0.14 |
| alpha band [8-12 Hz] oscillatory neurons (%) | 11.58 | 15.52 | 0.46 |
| beta band [12-30 Hz] oscillatory neurons (%) | 7.37 | 10.92 | 0.39 |
| gamma band [30-100 Hz] oscillatory neurons (%) | 12.63 | 16.09 | 0.48 |
| oscillatory neurons (%) | 41.05 | 48.28 | 0.30 |

**Supplementary Table 2 The neural features measured for GPi and GPe neurons.** Continuous metrics are presented as (median ± interquartile range). Dunn’s test with Holm-Bonferroni multiple comparison correction was used for continuous neural features, while Fisher's exact test was employed for binary neural features to compare GPi and GPe activity.
